# Impact of Designated Recovery Rehabilitation Institutions on the Readmission Rate of Older Adults

**DOI:** 10.3390/jcm15031009

**Published:** 2026-01-27

**Authors:** Kwang Bae Lee, Tae Hyun Kim, Sung-In Jang, Yun Seo Jang, Eun-Cheol Park

**Affiliations:** 1 R&D Center, Korea Medical Institute, Seoul 04522, Republic of Korea; 2 Department of Biohealth Industry, Graduate School of Transdisciplinary Health Sciences, Yonsei University, Seoul 03722, Republic of Korea; 3 Department of Healthcare Management, Graduate School of Public Health, Yonsei University, Seoul 03722, Republic of Korea; 4 Institute of Health Services Research, Yonsei University, Seoul 03722, Republic of Korea; 5 Department of Preventive Medicine, Yonsei University College of Medicine, Seoul 03722, Republic of Korea; 6 Institute of Health Insurance Research, National Health Insurance Service, Wonju 26464, Republic of Korea

**Keywords:** rehabilitation, central nervous system disorder, readmission, long-term disability, rehabilitation centers

## Abstract

**Background/Objectives**: With the global rise in chronic diseases among older adults, rehabilitation services have become essential, particularly for those with cerebrovascular and central nervous system (CNS) disorders, which lead to significant long-term disabilities. To determine the impact of designated rehabilitation medical institutions on the readmission rates of older patients with CNS disorders who receive surgical interventions. **Methods**: This was a population-based cohort study. Data was obtained from the National Health Insurance Service database (2002–2019). Fifteen designated institutions participated in the pilot project for convalescent rehabilitation. We analyzed the data of 1019 patients before and after the implementation of the designated rehabilitation institution. The study sample included (1) patients admitted to 15 designated institutions participating in the pilot project for convalescent rehabilitation and (2) patients diagnosed with conditions classified under the rehabilitation patient group, Rehabilitation Impairment Category 1 to 7. The intervention was the pilot project for designated rehabilitation institutions, launched in October 2017. The primary outcome of interest was the readmission rate of older patients with CNS disorders who received surgical interventions. Interrupted time series analysis with segmented regression was used to assess changes in the 30-day readmission rates. **Results**: Post-intervention, an 8% reduction in 30-day readmission rates (estimate, 0.9225; 95% confidence interval: 0.9129–0.9322, *p* < 0.0001) was observed. Subgroup analysis showed a significant decline in readmission rates across various patient groups, including those with disabilities, high Charlson Comorbidity Index scores, and extended hospital stays. The regions outside Seoul (capital city), particularly Gyeonggi/Incheon (areas around Seoul) and other areas (i.e., rural), also showed a significant decrease in readmission trends after the intervention. **Conclusions**: Designated rehabilitation medical institutions led to a significant reduction in readmission rates of older patients with CNS disorders, suggesting that these institutions effectively support recovery and reduce the burden of readmission for patients with severe conditions and those residing in non-capital cities.

## 1. Introduction

With the global population aging rapidly, the prevalence of chronic diseases in the older population is increasing, highlighting the ongoing need for rehabilitation-focused recovery healthcare services [1,2,3,4,5]. The main purpose of rehabilitation is to help patients reach the social return stage by reducing the rate of disease recurrence [6]. Cerebrovascular diseases, in particular, are known to have severe sequelae, making rehabilitation during the recovery phase after acute treatment crucial to minimize the difficulties of independent daily living caused by these disabilities [4,7,8,9]. The central nervous system (CNS), which includes the brain and spinal cord, is a critical part of the body and is often affected by cerebrovascular diseases [10]. These conditions can lead to various symptoms such as neuropathy, motor and sensory impairments, and psychological issues [11,12,13]. CNS disorders can involve serious complications such as stroke, cerebral infarction, Parkinson’s disease, and multiple sclerosis [14,15,16,17], all of which are chronic conditions requiring continuous treatment and management after surgery [18,19,20]. During the postoperative and recovery phases, patients often face multifaceted challenges beyond motor deficits, including mental and cognitive dysfunction, as well as systemic symptoms such as persistent fatigue, nausea, chronic headaches, and dizziness [21,22]. Furthermore, specific clinical issues such as swallowing problems (dysphagia), temporary confusion, and balance impairments significantly hinder daily functioning [23]. Early and appropriate rehabilitation leads to enhanced patient outcomes [24,25,26]. Especially for patients with CNS diseases, rehabilitation can enhance the quality of life, functioning, and independence by helping them adapt to and develop compensatory strategies [18,20,24].

Most older patients with chronic conditions require rehabilitation, and as medical expenses for older adults continue to rise, rehabilitation costs are also increasing [27]. In response, the government has established a recovery rehabilitation healthcare system aimed at minimizing disabilities and enabling early return to daily life through intensive rehabilitation during the functional recovery period following surgery or disease onset [28]. This system involves transferring patients who have undergone surgery or treatment in acute care hospitals, such as tertiary or general hospitals, and whose condition has stabilized but who still require consistent inpatient rehabilitation, to recovery rehabilitation medical institutions within a certain period. These institutions focus on providing intensive rehabilitation services geared toward social reintegration. Additionally, the government has designated recovery rehabilitation medical institutions in each region to ensure coordination and collaboration with community networks and teams.

The effectiveness of inpatient rehabilitation programs abroad has been quite significant. In Japan, patients with stroke admitted to the “Kaifukuki” rehabilitation ward, a recovery rehabilitation ward, showed improvements in their functional assessment scores, such as the Barthel index and functional independence measure, after receiving rehabilitation [29,30]. Similarly, in the United States, patients with cerebrovascular CNS disorders admitted to major rehabilitation facilities, such as inpatient rehabilitation facilities (IRFs), demonstrated improvements in motor function and an increased tendency to be discharged back into the community [31,32,33]. The demand for rehabilitation services was particularly high among older adult patients, with independent IRFs identified as a significant factor in cognitive recovery for older adults [33]. However, while the designation and operation of rehabilitation medical institutions began as a pilot project in 2017 and have been steadily implemented, leading to the main project launch in 2020, domestic studies comparing the effectiveness of rehabilitation services before and after the designation are lacking. Existing studies have primarily focused on differences in patient outcomes based on the utilization of rehabilitation services [34,35], rather than directly assessing the impact of the designation of rehabilitation medical institutions.

Therefore, this study aimed to investigate whether the rates of readmission after discharge of patients who underwent surgery for CNS disorders changed after the launch of designated rehabilitation medical institutions across different regions, utilizing population-based older adult cohort data. Through this analysis, the study seeks to determine whether intensive rehabilitation provided in designated recovery rehabilitation medical institutions has effectively reduced the likelihood of patient readmission and facilitated their return to the community.

## 2. Materials and Methods

### 2.1. Data

This study was conducted as a population-based cohort study utilizing the National Health Insurance Service (NHIS) database. The NHIS operates as a government-run social security system that provides extensive medical coverage to all citizens, except those eligible for medical aid. The NHIS offers researchers access to all claims data for academic research and policy development. This database includes health examination records, medical utilization claims, sociodemographic characteristics, and mortality information [36]. Notably, the claims database represents the largest dataset encompassing the entire medical history of the population. The NHIS-Senior cohort database we used consists of a randomly selected sample, including 558,147 adults aged ≥ 60 years [37]. This sample represents 10% of the population in the same age group [36,37]. The data collection period was 1 January 2002 to 31 December 2019, tracking individuals unless they died or emigrated, according to the National Health Insurance Act [36].

### 2.2. Study Population

**Figure 1:**
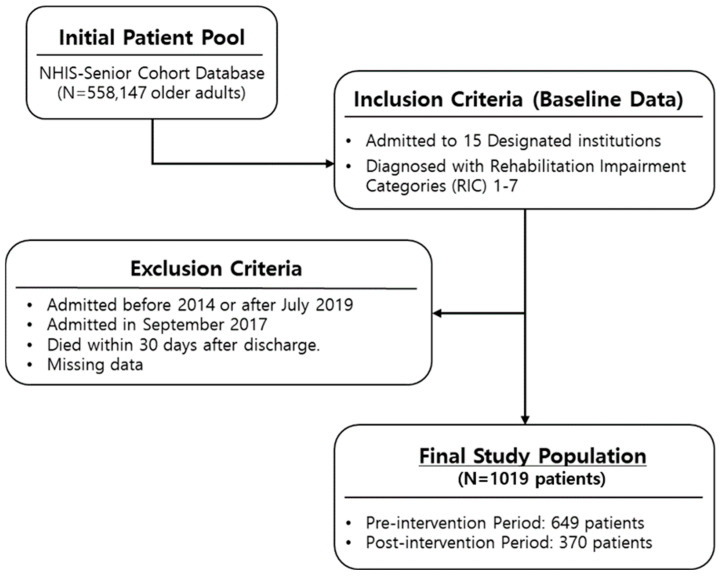
Flow diagram of the study.

The study sample included (1) patients admitted to 15 designated institutions participating in the pilot project for convalescent rehabilitation and (2) patients diagnosed with conditions classified under Rehabilitation Impairment Category diagnosis categories 1 to 7 (Table S1). To measure readmission as an outcome after discharge, the following exclusion criteria were applied to eliminate patients whose follow-up might have been interrupted: (1) patients who died within 30 days after discharge and (2) patients admitted after July 2019. Additionally, given the interrupted time series (ITS) design and the consideration of intervention dates, (1) patients whose first admission date was before 2014 and (2) patients admitted in September 2017 were also excluded. Only the first admission per patient ID was included in the analysis. After excluding patients with missing data, a total of 1019 patients were included in the study sample (Figure 1).

### 2.3. Variables

#### 2.3.1. Intervention

The intervention was defined as the pilot project for designated convalescent rehabilitation medical institutions launched on 1 October 2017. This project was established to provide intensive rehabilitation services during the functional recovery phase to minimize disability and facilitate early social reintegration. The core of the intervention involved a multidisciplinary team approach comprising rehabilitation physicians, nurses, physical and occupational therapists, and social workers who provided at least 2 to 3 h of intensive, customized therapy daily based on the patient’s functional status. The designation dates of the rehabilitation medical institutions were set as the reference point (time zero) for all subjects, with the period from 1 January 2015 to 30 September 2017 set as the pre-intervention period and the period from 1 October 2017 to 31 December 2019 set as the post-intervention period, aligning with the end date of the study data. Consequently, only 15 designated institutions involved in the pilot project were included in the study. These operating institutions implemented a systematic discharge planning process. To identify the implementation of these services, we utilized the following pilot project-specific billing codes: Integrated Planning and Management Fee for Convalescent Rehabilitation Pilot Institutions (IA810, IA811, IA820, IA821) and Integrated Rehabilitation Functional Assessment Fee (IA830, IA840).

#### 2.3.2. Outcome

The primary outcome of interest was the readmission rate. Specifically, to assess whether social reintegration was achieved, readmission was defined as readmission within 1 month after discharge from the rehabilitation medical institution due to the same diagnosis.

#### 2.3.3. Covariates

The following patient characteristics were included as covariates: sex (male, female), age (<65, 65–69, 70–74, 75–79, 80+ years), residential area (metropolitan, urban, rural), income level (low, middle, high), disability status (yes, no), length of stay (0–10, 11–30, 31–90, 91+ days), and Charlson Comorbidity Index (CCI) score (0–2, 3–4, 5+). Additionally, our analyses were adjusted for the following hospital characteristics: hospital location (Seoul, Gyeonggi and Incheon, others) and number of beds (categorized into quartiles). The CCI was utilized to assess the baseline health status and comorbidity burden of the participants. The CCI, based on comorbidities, was determined using International Classification of Diseases, 10th Revision, codes and involved assigning weights (1, 2, 3, or 6) to 19 comorbid conditions, such as congestive heart failure, diabetes, and malignancy, based on their clinical severity and relative 1-year mortality risk [38]. By summing these weights for each patient, we quantified the overall clinical complexity.

### 2.4. Statistical Analysis

Chi-square test was used to assess and compare the general characteristics of the study population. Descriptive statistics are presented as frequencies (N) and percentages (%). An ITS analysis with segmented regression was employed to examine time trends and changes in the outcomes. The ITS model, constructed with linear regression, included three time-related variables, with regression coefficients estimating the pre-intervention slope, level change at the time of intervention, and post-intervention slope change [39]. The slope change quantifies the difference between the pre- and post-intervention slopes, while the level change indicates the immediate effect of the intervention by showing the absolute change in outcomes at the time of intervention [36]. A log link function was applied in the generalized linear model for segmented regression, requiring model coefficients to be exponentiated to reflect trends and changes in outcomes on the original scale (E(Yi) = μi). To interpret the model coefficients, the log[E(Yi)] was converted into multiplicative terms to reflect the original scale. This model estimates the baseline level (i.e., intercept) with β0, initial trend (i.e., baseline trend) with β1, immediate effect of the intervention (i.e., level change after intervention) with β2, and trend change after the intervention with β3. The post-intervention trend (i.e., follow-up outcome trend) is captured by the sum of β1 and β3.

log(μi)  =  β0  +  β1  ×  timet  +  β2  ×  interventiont  +  β3  ×  time after interventiont  +  etc

Additionally, subgroup analysis was conducted to examine readmission stratified by disability status, CCI scores, lengths of stay, and location (region) of the medical institution. All analyses were adjusted for covariates, and the results are presented as parameter estimates with 95% confidence intervals (CIs). Statistical analyses were performed using SAS software version 9.4 (SAS Institute, Cary, NC, USA), with statistical significance set at *p* < 0.05. Statistical analysis was conducted between 12 July 2024 and 15 August 2024.

## 3. Results

Table 1 shows the general characteristics of the study population. Among the 1019 participants, a majority were male (55.54%), resided in rural areas (39.06%), and belonged to the low-income group (48.58%). Additionally, many patients had disabilities (86.10%) and high CCI scores (82.43%). The participant distribution rates before and after the “Convalescent Rehabilitation Medical Institution Designation and Operation Project” were 63.69% (649) and 36.31% (370), respectively. Compared to the pre-intervention period, the 30-day readmission rate decreased by 9.6% (pre-intervention: 45.3%, post-intervention: 35.7%), and this reduction was statistically significant as per the chi-square test result (*p* = 0.0027).

The segmented regression analysis conducted to estimate the probability of 30-day readmission for the same disease group after discharge, considering all covariates (Table 2), indicated that the likelihood of readmission decreased by 8% after the designation of convalescent rehabilitation medical institutions (estimate, 0.9225; 95% confidence interval [CI], 0.9129–0.9322; *p* < 0.0001). Although there was an increase in the readmission rate immediately after the intervention (estimate, 1.0618; 95% CI, 1.0140–1.1118; *p* = 0.0107), the overall readmission trend after the intervention showed a 6% decrease (estimate, 0.9418; 95% CI, 0.9330–0.9507; *p* < 0.0001).

**Figure 2:**
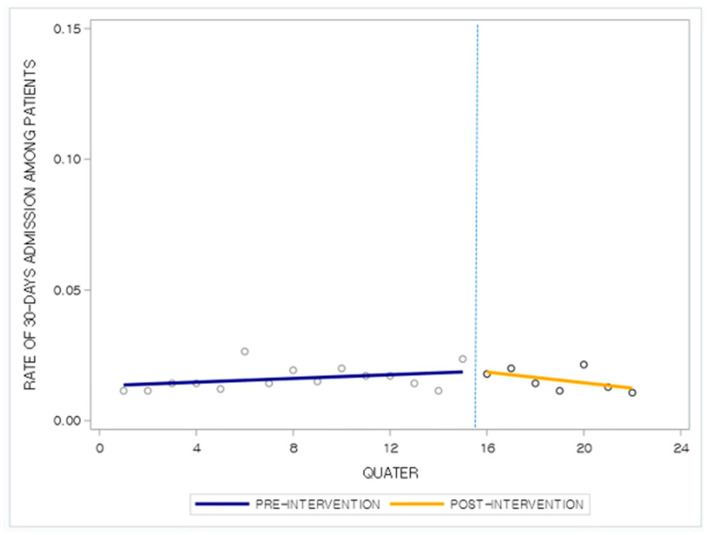
Factors associated with trends of readmission rate per designated rehabilitation medical institution.

As shown in Table 2, the parameter estimates allowed for a quantitative assessment of the magnitude of the level and trend changes, while Figure 2 intuitively illustrates the post-intervention outcome trends. The ITS results indicated that the probability of readmission within 30 days for the same disease group among patients with CNS disorders decreased after the launch of the “Convalescent Rehabilitation Medical Institution Designation Project.” As depicted in the figure, while the baseline trend showed an increase in the readmission risk (estimate, 1.0618; 95% CI, 1.0140–1.1118; *p* = 0.0107), a decreasing trend occurred after the intervention (estimate, 0.9225; 95% CI, 0.9129–0.9322; *p* < 0.0001).

Table 3 presents the results of subgroup analysis stratified by disability status, CCI score, length of stay, and medical institution region. All other covariates, except for the stratified ones, were statistically adjusted to calculate the estimates. Patients with disabilities (trend: before the intervention, 1.0147; *p* < 0.0001; after the intervention, 1.0965; *p* = 0.0011), those with a CCI score of 3 (trend: 1.0269; *p* < 0.0001; intervention: 1.0649; *p* = 0.0151), and those with a CCI score of 5 or greater (trend: before the intervention, 1.0212; *p* < 0.0001; after the intervention, 1.5437; *p* = 0.0305) showed an increasing trend in 30-day readmission rates before the intervention as well as immediately after the intervention. Additionally, patients with a length of stay exceeding 30 days (trend: before the intervention, 1.0194; *p* < 0.0001; after the intervention, 1.1428; *p* = 0.0023) or 90 days (trend: before the intervention, 1.0199; *p* < 0.0001; after the intervention, 1.0507; *p* = 0.0404) also showed statistically significant increments in 30-day readmission rates both before and immediately after the intervention. However, follow-up results indicated that both the trend and the follow-up trend in 30-day readmission rates significantly decreased in these groups after the intervention: For patients with disabilities (trend after intervention: 0.9266; *p* < 0.0001; trend at follow-up: 0.9402; *p* < 0.0001), those with a CCI score of 3 or higher (trend after intervention: 0.9225; *p* < 0.0001; trend at follow-up: 0.9473; *p* < 0.0001) or a CCI score of 5 or higher (trend after intervention: 0.9223; *p* < 0.0001; trend at follow-up: 0.9419; *p* < 0.0001), and those with a length of stay exceeding 30 days (trend after intervention: 0.9076; *p* < 0.0001; trend at follow-up: 0.9252; *p* < 0.0001) or exceeding 90 days (trend after intervention: 0.9508; *p* < 0.0001; trend at follow-up: 0.9696; *p* = 0.0003), the readmission trends after the intervention and follow-up both showed a statistically significant decrease after the intervention.

Finally, when the hospital regions were analyzed, we found that in regions other than Seoul, specifically Gyeonggi/Incheon and other regions, the readmission trend before the intervention was increasing (Gyeonggi & Incheon: trend estimate, 1.0205; *p* < 0.0001; intervention estimate, 1.0651; *p* = 0.1413; others: trend estimate, 1.0211; *p* < 0.0001; intervention estimate, 1.0532; *p* = 0.1122). However, immediately after the intervention, no significant association was observed, but the readmission trends after the intervention and follow-up showed a significant decrease (Gyeonggi & Incheon: trend after intervention estimate, 0.9111; *p* < 0.0001; follow-up trend estimate, 0.9298; *p* < 0.0001; others: trend after intervention estimate, 0.9333; *p* < 0.0001; follow-up outcome trend estimate, 0.9409; *p* < 0.0001).

## 4. Discussion

In this study, we examined whether there was a difference in the trend of 30-day readmission rates for the same disease before and after the launch of designated rehabilitation medical institutions. Our findings show that the implementation of the convalescent rehabilitation medical institution designation pilot project significantly lowered the likelihood of 30-day readmission for patients with CNS disorders, with an 8% decrease in rates following the intervention, despite an initial rise immediately after the project’s start. Subgroup analysis highlighted that patients with high CCI scores, long hospital stays, and those living outside of Seoul, particularly in Gyeonggi/Incheon or other cities, saw significant reductions in readmission trends after the intervention. These results emphasize the effectiveness of designated rehabilitation institutions in reducing readmission risks and improving patient outcomes. Taken together, this 8% reduction indicates that the pilot designation of rehabilitation medical institutions successfully transformed a previously fragmented post-acute care pathway into a more coordinated system, in which older adults with CNS disorders are less likely to return to acute hospitals and more likely to complete a structured recovery trajectory.

Most previous studies have shown that older adult patients with cardiovascular and CNS diseases have enhanced outcomes when they receive rehabilitation after surgery [19,24,25,26], supporting our findings that designating specific medical institutions as convalescent rehabilitation centers, enabling focused rehabilitation management, is effective for the social reintegration of patients who undergo surgery. This mechanism involves encouraging patients who have undergone surgery at tertiary or general hospitals to be admitted to designated rehabilitation institutions for consistent rehabilitation within a certain period after surgery [40], considering that it can be challenging for patients who undergo surgery to maintain consistent outpatient rehabilitation after discharge due to accessibility issues [41,42]. Our findings extend this evidence by suggesting that not only the provision of rehabilitation itself, but also the formalization of referral pathways and institutional roles through a national designation policy, can be a decisive factor in ensuring that patients actually receive timely and continuous rehabilitation rather than being lost in the transition from acute to post-acute care.

As highlighted by our results, the trend of increasing readmissions before the policy introduction in regions outside of Seoul (capital city), such as Gyeonggi/Incheon (areas around Seoul) and other areas (i.e., rural), was reversed after the intervention, indicating that the policy had a positive impact, particularly in non-metropolitan areas. This phenomenon can be attributed to the challenges faced by patients living in rural areas, where the distance between their homes and the hospitals where they had surgery makes it difficult for them to maintain rehabilitation [42,43]. Additionally, older patients who have undergone surgery often find it difficult to travel long distances to receive treatment on their own [43]. Therefore, receiving rehabilitation at hospitals that are connected to their place of residence can be beneficial, underscoring the importance of the role played by the designated rehabilitation medical institutions in each region, particularly for older adult patients living in areas with limited access to rehabilitation services. From a policy perspective, this suggests that placing designated rehabilitation institutions closer to where older adults live is not merely a matter of convenience, but a structural intervention that can reverse adverse trends in rehospitalization and narrow regional health disparities in outcomes for CNS disorders.

Moreover, for patient groups with disabilities, multiple comorbidities, or long hospital stays, an increasing trend in readmissions was observed before the designation of rehabilitation medical institutions. However, after the designation, a decrease in readmissions was observed, aligning with previous research findings, emphasizing the importance of rehabilitation for patients with severe conditions [44,45]. In clinical terms, this means that those who are most likely to cycle repeatedly between home and hospital may benefit the most when rehabilitation is delivered within a clearly defined, multidisciplinary framework, and that prioritizing these high-risk groups within designated institutions could yield substantial gains in both patient outcomes and system efficiency. Nevertheless, the increase in readmissions immediately after the pilot policy’s introduction could be attributed to the initial lack of a well-established system, making the policy less effective for patients with severe conditions who required intensive rehabilitation [46]. Therefore, further research is needed to evaluate the long-term effects of the stabilized implementation of this policy.

Overall, the vertical sequence from our findings, reduced 30-day readmissions, associations in high-risk and non-metropolitan groups, and sustained post-intervention trend changes, supports the view that designated rehabilitation institutions should be further expanded and refined as a core component of national strategies to improve recovery, equity, and long-term sustainability in the care of older adults with CNS disorders. Given the policy-oriented nature of this study, it is also important to consider the health economic implications of designated recovery rehabilitation institutions. A previous study reported that the average total economic burden per stroke patient in Korea was approximately 7.9 million KRW (about USD 7247), with direct medical costs accounting for a substantial proportion [47]. Other reports indicate that the cost of an inpatient rehabilitation episode typically ranges from roughly 3 to 5 million KRW per patient, depending on the intensity and duration of rehabilitation services [48,49]. When benchmarks are considered together with our finding of an 8% reduction in 30-day readmission rates, it is plausible that the designated rehabilitation institution policy yields meaningful savings for the national health insurance system. Preventing even a modest number of readmissions can avoid high acute care costs associated with surgery, hospitalization, and follow-up care. From a health economics perspective, the designated rehabilitation institution model may therefore function as a cost-effective intervention that improves clinical outcomes while contributing to the containment of national healthcare expenditure and more efficient resource allocation. Future studies using detailed cost data are warranted to quantify the net economic impact of this policy more precisely.

### 4.1. Advantages and Disadvantages

There are some disadvantages to this study. First, as this analysis was based on administrative claims data, individual clinical and functional outcomes such as global disability or cognitive and motor recovery assessed through validated scales (e.g., modified Rankin Scale, MMSE, or MRC scale) could not be evaluated. Second, although the designated rehabilitation institutions are organized with multidisciplinary teams including physiatrists, nurses, physical therapists, occupational therapists, and social welfare workers, the NHIS database does not contain detailed information on team composition or the proportion of each professional’s involvement in rehabilitation delivery. Therefore, the impact of specific professional contributions could not be analyzed. Third, the use of medical claims data meant that baseline health status and other related variables could not be accounted for, although the CCI score was adjusted. Residual confounding from unmeasured variables remains a possibility. Fourth, administrative claims data have inherent limitations, as they are designed for payment processing rather than providing a complete medical record, potentially leading to inaccuracies in disease coding. Fifth, the introduction of the rehabilitation institution designation policy might have coincided with other healthcare changes, which we could not control, possibly leading to an overestimation of the results. Therefore, we tried to minimize bias by specifying and analyzing the data of only patients who underwent surgery and needed rehabilitation.

Despite these disadvantages, the study has significant advantages. The national NHIS database provided comprehensive data covering the entire population, allowing for robust and generalizable findings. Additionally, the ITS design, a strong quasi-experimental method, effectively captured longitudinal trends and changes before and after the intervention, offering a thorough analysis compared to that in previous studies. While many studies have examined the importance and effectiveness of rehabilitation for patients with cerebrovascular and CNS conditions, to our knowledge, no study has explored the impact of the introduction of designated rehabilitation medical institutions. Our results underscore not only the significance of rehabilitation but also the need for concerted efforts by the government and medical institutions to ensure that patients can consistently receive rehabilitation care.

### 4.2. Practical Implications

Our findings offer several practical implications for healthcare policy and clinical practice. First, the significant reduction in 30-day readmission rates highlights the effectiveness of the designated rehabilitation system; therefore, policymakers should consider expanding these institutions to ensure regional health equity, particularly in non-metropolitan areas. Second, the results emphasize the importance of early and intensive rehabilitation for high-risk groups, such as patients with high comorbidity burdens or long hospital stays. Clinically, this suggests that a structured, multidisciplinary approach in a specialized environment is a critical factor in preventing costly readmissions and facilitating successful social reintegration for older adults with CNS disorders.

## 5. Conclusions

In conclusion, our findings suggest that the introduction of designated recovery rehabilitation medical institutions in each region, as part of government health policy, led to a significant 8% reduction in 30-day readmission rates among older patients with cerebrovascular and CNS diseases who underwent surgery in tertiary or general hospitals. This finding supports the current research evidence on the effectiveness of consistent management and rehabilitation for patients who undergo surgery. Furthermore, since this policy has been particularly effective in high-risk and non-metropolitan areas, it should be considered to expand and accurately distinguish the areas covered by these agencies. Such efforts will help enhance healthcare accessibility and reduce the medical burden for older adult patients with severe conditions, ultimately facilitating their successful social reintegration.

## Figures and Tables

**Table 1:** Characteristics of the study population.

Variables	Total		Readmission Within 30 Days				*p*-Value
			Yes		No		
			N	%	N	%	
Total	1019	100.00	426	41.8	593	58.2	
Designation of Rehabilitation Medical Institutions							0.0027
Pre-intervention	649	63.69	294	45.3	355	54.7	
Post-intervention	370	36.31	132	35.7	238	64.3	
Sex							0.0133
Male	566	55.54	256	45.2	310	54.8	
Female	453	44.46	170	37.5	283	62.5	
Age							0.0347
<65	293	28.75	145	49.5	148	50.5	
65–69	180	17.66	73	40.6	107	59.4	
70–74	186	18.25	72	38.7	114	61.3	
75–79	176	17.27	66	37.5	110	62.5	
≥80	184	18.06	70	38.0	114	62.0	
Region							0.3112
Metropolitan	255	25.02	98	38.4	157	61.6	
Urban	366	35.92	163	44.5	203	55.5	
Rural	398	39.06	165	41.5	233	58.5	
Income level							0.0069
High	258	25.32	127	49.2	131	50.8	
Middle	266	26.10	114	42.9	152	57.1	
Low	495	48.58	185	37.4	310	62.6	
Disability status							<0.0001
None	405	39.74	132	32.6	273	67.4	
Have	614	60.26	294	47.9	320	52.1	
Charlson Comorbidity Index							0.0242
0–2	36	3.53	8	22.2	28	77.8	
3–4	143	14.03	54	37.8	89	62.2	
≥5	840	82.43	364	43.3	476	56.7	
Lengths of stay							0.0016
0–10	118	11.58	67	56.8	51	43.2	
11–30	204	20.02	71	34.8	133	65.2	
31–90	319	31.31	129	40.4	190	59.6	
91–	378	37.10	159	42.1	219	57.9	
Medical institution region							0.0069
Seoul	81	7.95	34	42.0	47	58.0	
Gyeonggi, Incheon	342	33.56	120	35.1	222	64.9	
Others	596	58.49	272	45.6	324	54.4	
Number of hospital beds							<0.0001
Q1 (low)	340	33.37	136	40.0	204	60.0	
Q2	187	18.35	61	32.6	126	67.4	
Q3	276	27.09	98	35.5	178	64.5	
Q4 (high)	216	21.20	131	60.6	85	39.4	

**Table 2:** Interrupted time series analysis of the designation impact of rehabilitation medical institutions on the 30-day readmission after discharge of patients.

Variables	30-Day Readmission After Discharge		
	Exp(β)	95% CI	*p*-Value
Intercept (β0)	0.0000	(0.0000–0.0000)	<0.0001
Baseline trend (β1)	1.0210	(1.0162–1.0257)	<0.0001
Level change after intervention (β2)	1.0618	(1.0140–1.1118)	0.0107
Trend change after intervention (β3)	0.9225	(0.9129–0.9322)	<0.0001
Follow-up outcome trend (β1 + β3)	0.9418	(0.9330–0.9507)	<0.0001
Sex			
Male	1.0000		
Female	0.9585	(0.9290–0.9891)	0.0081
Age			
<65	1.0000		
65–69	0.9740	(0.9329–1.0170)	0.2317
70–74	0.9688	(0.9245–1.0152)	0.1836
75–79	0.9906	(0.9425–1.0411)	0.7090
≥80	0.9705	(0.9257–1.0176)	0.2153
Region			
Metropolitan	1.0000		
Urban	0.9882	(0.9389–1.0402)	0.6510
Rural	0.9968	(0.9493–1.0466)	0.8966
Income level			
High	1.0000		
Middle	1.0028	(0.9654–1.0417)	0.8858
Low	1.0164	(0.9810–1.0531)	0.3683
Disability status			
None	1.0000		
Have	0.9801	(0.9475–1.0138)	0.2443
Charlson Comorbidity Index			
0–2	1.0000		
3–4	0.9944	(0.9214–1.0731)	0.8844
≥5	0.9949	(0.9276–1.0670)	0.8850
Lengths of stay			
0–10	1.0000		
11–30	0.9357	(0.8710–1.0052)	0.0692
31–90	0.9241	(0.8603–0.9926)	0.0305
91–	0.9161	(0.8556–0.9810)	0.0121
Medical institution region			
Seoul	1.0000		
Gyeonggi, Incheon	1.0176	(0.9676–1.0703)	0.4972
Others	1.0388	(0.9783–1.1030)	0.2140
Number of hospital beds			
Q1 (low)	1.0000		
Q2	1.0456	(0.9791–1.1167)	0.1837
Q3	1.0076	(0.9688–1.0479)	0.7059
Not included	1.0047	(0.9611–1.0502)	0.8368

**Table 3:** Results of subgroup analysis stratified by dependent variables.

	30-Day Readmission After Discharge							
	Trend		Intervention		Trend After Intervention		Follow-Up Outcome Trend	
	Estimate (95% CI)	*p*-Value	Estimate (95% CI)	*p*-Value	Estimate (95% CI)	*p*-Value	Estimate (95% CI)	*p*-Value
**Disability status**								
None	1.0251 (1.0197–1.0305)	<0.0001	1.0300 (0.9794–1.0832)	0.2501	0.9153 (0.9045–0.9263)	<0.0001	0.9383 (0.9281–0.9487)	<0.0001
Have	1.0147 (1.0088–1.0207)	<0.0001	1.0965 (1.0373–1.1591)	0.0011	0.9266 (0.9152–0.9381)	<0.0001	0.9402 (0.9301–0.9504)	<0.0001
**Charlson Comorbidity Index**								
0–2	1.0112 (0.9887–1.0343)	0.3315	0.9695 (0.8425–1.1156)	0.6651	0.8826 (0.8223–0.9473)	0.0005	0.8925 (0.8314–0.9581)	0.0017
3–4	1.0269 (1.0136–1.0404)	<0.0001	1.0649 (1.0123–1.1203)	0.0151	0.9225 (0.8982–0.9475)	<0.0001	0.9473 (0.9250–0.9702)	<0.0001
≥5	1.0212 (1.0161–1.0264)	<0.0001	1.5437 (1.0416–2.2876)	0.0305	0.9223 (0.9116–0.9331)	<0.0001	0.9419 (0.9321–0.9518)	<0.0001
**Lengths of stay**								
0–10	1.0178 (0.9999–1.0360)	0.0520	0.9763 (0.8079–1.1797)	0.8035	0.9152 (0.8825–0.9492)	<0.0001	0.9315 (0.9024–0.9615)	<0.0001
11–30	1.0319 (1.0232–1.0407)	<0.0001	0.9943 (0.9107–1.0857)	0.8989	0.9011 (0.8834–0.9192)	<0.0001	0.9299 (0.9128–0.9472)	<0.0001
31–90	1.0194 (1.0112–1.0276)	<0.0001	1.1428 (1.0489–1.2453)	0.0023	0.9076 (0.8926–0.9229)	<0.0001	0.9252 (0.9116–0.9390)	<0.0001
91–	1.0199 (1.0130–1.0268)	<0.0001	1.0507 (1.0058–1.1313)	0.0404	0.9508 (0.9337–0.9681)	<0.0001	0.9696 (0.9537–0.9858)	0.0003
**Medical institution region**								
Seoul	1.0186 (0.9987–1.0388)	0.0667	1.1414 (0.9417–1.3834)	0.1778	0.9199 (0.8863–0.9547)	<0.0001	0.9370 (0.9048–0.9702)	0.0003
Gyeonggi, Incheon	1.0205 (1.0123–1.0287)	<0.0001	1.0651 (0.9792–1.1586)	0.1413	0.9111 (0.8957–0.9268)	<0.0001	0.9298 (0.9164–0.9434)	<0.0001
Others	1.0211 (1.0150–1.0272)	<0.0001	1.0532 (0.9880–1.1227)	0.1122	0.9333 (0.9203–0.9465)	<0.0001	0.9530 (0.9409–0.9652)	<0.0001

## Data Availability

Data is unavailable since only individual researchers authorized by the Korea National Health Insurance Service (http://nhiss.nhis.or.kr, accessed on 12 March 2024) can access the National Health Information Database.

## Supplementary Materials

The following supporting information can be downloaded at https://www.mdpi.com/article/10.3390/jcm15031009/s1. Table S1: Distribution of the study population.

## Abbreviations

NHIS	National Health Insurance Service
CNS	Central Nervous System
IRFs	Inpatient Rehabilitation Facilities
ITS	Interrupted Time Series
CCI	Charlson Comorbidity Index
