# Supplementary material for: Impact of Designated Recovery Rehabilitation Institutions on the Readmission Rate of Older Adults
Source: J Clin Med. 2026 Jan 27;15(3):1009. doi: 10.3390/jcm15031009 (PMC12898560; doi:10.3390/jcm15031009)
Supplement: Supplementary file 1 [file jcm-15-01009-s001.zip › jcm-4062776-supplementary.pdf]

Journal of
Clinical Medicine

MDPI

Supplementary Material

Table S1. Distribution of the study population

Variables	Total		Rehabilitation Medical Institutions				P-value
			Pre-designated		Post-designated		
			N	%	N	%	
**Total**	1,019	100.00	649	63.7	370	36.3	
Sex							0.9493
Male	566	55.54	360	55.5	206	55.7	
Female	453	44.46	289	44.5	164	44.3	
Age							<.0001
< 65	293	28.75	216	33.3	77	20.8	
65-69	180	17.66	110	16.9	70	18.9	
70-74	186	18.25	117	18.0	69	18.6	
75-79	176	17.27	112	17.3	64	17.3	
≥ 80	184	18.06	94	14.5	90	24.3	
Region							0.0002
Metropolitan	255	25.02	135	20.8	120	32.4	
Urban	366	35.92	246	37.9	120	32.4	
Rural	398	39.06	268	41.3	130	35.1	
Income level							0.3912
High	258	25.32	171	26.3	87	23.5	
Middle	266	26.10	173	26.7	93	25.1	
Low	495	48.58	305	47.0	190	51.4	
Disability status							0.9941
None	405	39.74	258	63.7	147	36.3	
Have	614	60.26	391	63.7	223	36.3	
Charlson Comorbidity index							0.0032
0-2	36	3.53	24	66.7	12	33.3	
3-4	143	14.03	73	51.0	70	49.0	
≥ 5	840	82.43	552	65.7	288	34.3	
Lengths of stay							0.0182
0-10	118	11.58	89	13.7	29	7.8	
11-30	204	20.02	119	18.3	85	23.0	
31-90	319	31.31	206	31.7	113	30.5	
91-	378	37.10	235	36.2	143	38.6	
Medical institution region							<.0001
Seoul	81	7.95	38	5.9	43	11.6	
Gyeonggi, Incheon	342	33.56	203	31.3	139	37.6	
Others	596	58.49	408	62.9	188	50.8	
Number of Hospital bed							0.0227
Q1(low)	340	33.37	201	31.0	139	37.6	
Q2	187	18.35	124	19.1	63	17.0	
Q3	276	27.09	170	26.2	106	28.6	
Q4(high)	216	21.2	154	23.7	62	16.8	

https://doi.org/10.3390/jcm15031009

https://doi.org/10.3390/jcm15031009
